# Consensus on the management of platinum-sensitive high-grade serous epithelial ovarian cancer in Lebanon

**DOI:** 10.1016/j.gore.2023.101186

**Published:** 2023-04-23

**Authors:** Reem Abdallah, David Atallah, Nizar Bitar, Georges Chahine, Hady Ghanem, Marwan Ghosn, Joseph Kattan, Fadi Nasr, Joseph Makdessi, Ali Shamseddine

**Affiliations:** a Department of Obstetrics and Gynecology, Division of Gynecologic Oncology, American University of Beirut Medical Center, Beirut, Lebanon; b Department of Obstetrics and Gynecology, Saint Joseph University Hospital-Hôtel-Dieu de France, Beirut, Lebanon; c Department of Internal Medicine, Division of Hematology-Oncology, Sahel General Hospital, Beirut, Lebanon; d Department of Hematology Oncology, Hôtel-Dieu de France – Saint Joseph University Hospital, Beirut, Lebanon; e Department of Internal Medicine, Division of Hematology/Oncology, Lebanese American University Medical Center – Rizk Hospital, Beirut, Lebanon; f Hematology Oncology Department, Faculty of Medicine, Saint Joseph University, Beirut, Lebanon; g Department of Internal Medicine, Division of Hemato-Oncology, Saint George Hospital-University Medical Center, Beirut, Lebanon; h Department of Internal Medicine, Division of Hematology-Oncology, American University of Beirut Medical Center, Beirut, Lebanon

**Keywords:** High-grade ovarian cancer, BRCA mutation, HRD, Cytoreductive surgery, PARP inhibitors, Practical guidance

## Abstract

Ovarian cancer is the most lethal gynecologic cancer. The high grade serous epithelial (HGSE) subtype is the most aggressive and it often presents at advanced stages, while screening programs have not proven beneficial. Management of the advanced stages (FIGO III and IV), which constitute the majority of diagnoses, usually consists of platinum-based chemotherapy and cytoreductive surgery (primary or interval) followed by maintenance therapy. Currently, the standard-of-care for advanced newly diagnosed HGSE ovarian cancer, as per international medical societies, starts with upfront cytoreductive surgery, followed by platinum-based chemotherapy (mostly carboplatin and paclitaxel) and/or anti-angiogenic agent bevacizumab, then maintenance therapy with a poly(ADP-ribose) polymerase (PARP) inhibitor with/without/or bevacizumab (continued). PARP inhibitor use depends on the patient’s genetic signature, mainly the breast cancer gene (BRCA) mutation and the homologous recombination deficiency (HRD) status. Therefore, genetic testing is recommended at diagnosis to inform treatment and prognosis.

In line with the evolving standard-of-care for ovarian cancer, a panel of experts in treating advanced ovarian cancer convened to lay down practical recommendations on the management of advanced ovarian cancer in Lebanon; since the currently applicable guidelines by the Lebanese Ministry of Public Health for cancer treatment have not been updated yet to reflect the treatment paradigm shift brought upon by the development and approval of PARP inhibitors. The current work reviews the leading clinical trials on PARP inhibitors (as maintenance for newly diagnosed advanced and platinum-sensitive relapsed ovarian cancer), presents international recommendations, and proposes treatment algorithms for optimal local practice.

## Highlights

• A multi-disciplinary team approach is recommended to optimize the management of Ovarian cancer.
• Primary cytoreduction by experts is advised; if unfeasible to opt for neoadjuvant therapy then interval debulking surgery.
• PARP inhibitors are recommended as a standard of care maintenance therapy in newly diagnosed advanced ovarian cancer.
• Early genetic testing is primordial and helps dictate treatment decisions in newly diagnosed advanced ovarian cancer.
• PARP inhibitors are recommended as maintenance therapy for platinum-sensitive relapse regardless of BRCA mutation status.

## 1 Introduction

Ovarian cancer is the leading cause of deaths among all gynaecological cancers (Sung et al., 2021). It is the 8th most common cancer among women worldwide with an incidence of 3.4% (International Agency for Research on Cancer, 2021) and the mortality rate is around 6.7% (International Agency for Research on Cancer, 2021). In 2020 only; about 313; 959 females were newly diagnosed with ovarian cancer (Sung et al., 2021). In Lebanon; according to the Global Cancer Observatory; published by the International Agency for Research on Cancer of the World Health Organization, the 2020 incidence of ovarian cancer in Lebanon was 1.8%, the mortality rate was 2.3% and the five-year prevalence was 16.05 per 100,000 women (International Agency for Research on Cancer, 2021). According to data from the National Cancer Registry; the crude ovarian cancer rate has fluctuated between 9.3 and 6.8 per 100,000 women in Lebanon between 2005 and 2016, with the highest incidence recorded after the age of 45 years (Ministry of Public Health. National Cancer Registry NCR. Lebanon, 2015).

Ovarian cancer is a heterogeneous disease consisting of many distinct subtypes of which the high-grade serous epithelial (HGSE) histological subtype, known as type II tumor, is the most common and presents a high rate of relapse with close to 80% of patients relapsing despite initial response to surgery and chemotherapy (Gee et al., 2018). HGSE ovarian cancer is an aggressive cancer that is typically diagnosed at advanced stages and is the most lethal (Gee et al., 2018, Hoppenot et al., 2018, Narod, 2016). According to Surveillance, Epidemiology and End Results (SEER) programs, the 5-year survival rate for patients with ovarian cancer was estimated at 49.7% in 2018 and the proportionate mortality from ovarian cancer stood at 2.1% in 2022 (SEER Ovarian Cancer, 2018). The 10-year survival of patients diagnosed with early-stage HGSE is 55%, compared to only 15–30% for patients with advanced-stage disease at diagnosis (Gee et al., 2018, Hoppenot et al., 2018, Narod, 2016).

Interestingly, HGSE ovarian cancers harbor the *TP53* mutation, but this has not been correlated to any clinical implication. Another gene of interest in HGSE ovarian cancer, the breast cancer gene (BRCA), plays a role in DNA damage repair via homologous recombination (HR). Indeed, mutation of this gene (*BRCAm*) leads to HR deficiency (HRD), conferring a prediction of response to both platinum (Petrucelli et al., 2010) and poly(ADP-ribose) polymerase (PARP) inhibitor therapies (Chen, 2011) and this has been shown to have an essential clinical implication through a targeted management plan.

Hence, most medical societies have recently updated their guidelines to include PARP inhibitors and precision medicine into their management algorithms based on the unprecedented results of the trials, reviewed below (Moore et al., 2018, Ray-Coquard et al., 2019, González-Martín et al., 2019, Coleman et al., 2019). In Lebanon, the National Cancer Treatment Guidelines of the Lebanese Ministry of Public Health have not been updated yet to include currently applicable evidence-based guidelines for the treatment of ovarian cancer (Committee, 2018). However, PARP inhibitors are currently registered and prescribed through individualized clinician-based decisions.

The objective of this consensus is to foster a national expert opinion with the aim of updating, optimizing and standardizing the practices and to underline the importance of a multidisciplinary team (MDT) approach and of genetic testing in the management pathways thus improving the quality of care of patients.

## 2 Methods

A panel of experts including members of the Lebanese Society of Medical Oncology (LSMO) and the Lebanese Gynecology Oncology Group (LGOG) have convened and discussed recent updates in the management of newly diagnosed advanced ovarian cancer as well as platinum sensitive relapsed ovarian cancer.

It was chaired by Pr. S. Pignata, member of the educational committee of the European Society of Medical Oncology (ESMO) and past president of the European Network of Gynecological Oncological Trial Groups (ENGOT). International guidelines and key trials (up until December 2021) were presented and discussed as well as worldwide management, genetic testing implementation and MDT approach strategies. Panelists discussed the trials and international practice in ovarian cancer as well as local real-world practice and they established national recommendations. Global guidelines (American Society of Clinical Oncology [ASCO], ESMO/European Society of Gynaecologial Oncology [ESMO/ESGO], British Gynaecological Cancer Society [BGCS] and National Comprehensive Cancer Network [NCCN]) and literature-based latest medical evidence are also displayed in this joint statement.

In particular, we review the multidisciplinary approach, treatment pillars and international recommendations in (A) newly diagnosed advanced HGSE ovarian cancer; followed by (B) genetic testing for BRCA and HRD globally, and recommendations for local practice; and (C) management of platinum-sensitive relapsed ovarian cancer.

## 3 Newly diagnosed advanced HGSE ovarian cancer

The traditional standard of care for newly diagnosed advanced HGSE ovarian cancer (stage III and stage IV according to The International Federation of Gynecology and Obstetrics [FIGO]) includes cytoreductive surgery and platinum-based chemotherapy with or without concurrent and maintenance bevacizumab (Banerjee et al., 2020). Since 2018, PARP inhibitors were incorporated as the standard-of-care maintenance therapy for patients with complete or partial response to surgery and chemotherapy since despite the high rates of response to first-line therapy more than 70% of patients relapse within 3 years (Committee, 2018). Below is a detailed description of treatment pillars, which are currently valid for first-line management of newly diagnosed HGSE ovarian cancer.

### 3.1 Multidisciplinary team approach

International guidelines mostly recommend a multidisciplinary team (MDT) approach as a determinant of prognosis and quality of life benefits to patients.

• The ASCO recommends a MDT approach to all cancer patients in general, and that all women with advanced invasive epithelial ovarian cancer should be evaluated by a gynecologist oncologist prior to therapy initiation to determine whether they are candidates for primary cytoreductive surgery (Vanderpuye et al., 2021, Wright et al., 2016).
• The ESMO/ESGO joint consensus states that both diagnostic work-up and treatment must be carried out in a MDT setting and in a specialist ovarian cancer center. It recommends that patient selection for primary cytoreductive surgery or for neoadjuvant treatment be carried out by a MDT in a specialist ovarian care center (Colombo et al., 2019).
• The BGCS clearly states that a MDT discussion within a quorate MDT is fundamental to the appropriate management of each individual patient and to be clearly described in an operational document. In addition, it also stipulates that treating oncologists should be core members of a specialist MDT and that surgery must take place in a specialized cancer center by specialized surgeons who are core members of the MDT (Fotopoulou et al., 2017).

Furthermore, a recent article by Falzone *et al.* showcased the differences between the traditional linear approach in the management of ovarian cancer (where the surgeon excises the tumor, the pathologist analyzes it and the medical oncologist designs the pharmacological treatment) *versus* the optimized recent shift into a circular approach (where all three medical specialists interact together and discuss the optimal patient-centered therapeutic management) thus resulting in an improvement in prognosis, management outcomes and quality of life (Falzone et al., 2021). Communication through MDT is essential for better prognosis.

All panelists uniformly agreed and aligned on the importance of MDT. They recommend MDT discussion of all advanced ovarian cancer cases and raise attention to the need to increase awareness on the national level on the improvement in care and in all related outcomes brought by a MDT approach involving ovarian cancer specialists. The MDT should include primarily an oncologist, a radiologist, a pathologist (molecular pathologist if possible) and a gynecologic cancer surgeon, in addition to different other members. Other specialties might be included (specialist nurses, stoma-therapists, nutritionists, radiotherapists, or onco-geneticists). To cope with the challenges in implementing such an approach at the country scale, especially in peripheral centers, panelists underline the need for national awareness campaigns as well as monthly national tumor boards for discussions of all cases and for structured decisions. This can be facilitated through networking and the establishment of an online platform. It will also increase overall expertise and communication. They also expressed their willingness to participate regularly to such boards if needed. Finally, they all agreed that cytoreductive surgeries must be performed by expert gynecologic cancer surgeons, as recommended by all societies including NCCN.

• Optimal management of advanced ovarian cancer should be dictated by a MDT.
• A MDT should include primarily an oncologist, a radiologist, a pathologist, and an expert gynecologic cancer surgeon.
• Importance of national MDT awareness campaigns, in particular for peripheral practices.
• Need for national monthly tumor boards to discuss all cases with the participation of experts from different institutions.
• Networking and online platforms are currently the practical national MDT organizational tools.

### 3.2 Treatment pillars

#### 3.2.1 Role and timing of surgery

It is clear, as has been shown by most studies, that optimal cytoreduction tremendously affects prognosis and that the volume of residual disease after primary debulking surgery is directly correlated with survival benefits (Lyons et al., 2020). Complete resection of all macroscopic disease has been shown to be the single most important independent prognostic factor in advanced epithelial ovarian cancer (Colombo et al., 2019). The aim should be no gross residual disease and efforts towards this outcome must be maximized. The main and primary decision is therefore whether surgery is feasible upfront or following neoadjuvant chemotherapy (interval debulking surgery) (Lyons et al., 2020). International guidelines favor primary debulking surgery as a priority whenever feasible and recommend that it should be performed by a gynecological oncological surgeon (Wright et al., 2016, Colombo et al., 2019, Fotopoulou et al., 2017, Armstrong et al., 2021, Querleu et al., 2016). Evaluation should be conducted by the latter amidst a MDT and the decision should be based on the predicted ability of reaching a complete cytoreduction (R0). If it is not feasible, as judged by the expert surgeon, neoadjuvant chemotherapy is preferable followed by interval debulking surgery. Expert surgeons may also evaluate resectability through laparoscopic procedures.

• The NCCN prioritizes primary debulking surgery as the recommended approach for advanced-stage disease if the patient is a surgical candidate, optimal cytoreduction appears feasible and fertility is not a concern (Armstrong et al., 2021). Fundamentally, evaluation by a gynecologist oncologist is recommended for all patients with suspected ovarian malignancies and primary assessment as well as debulking by a gynecologist oncologist confers a survival advantage (Armstrong et al., 2021).
• The ESMO/ESGO guidelines state that when complete surgery with no macroscopic visible disease appears feasible (both spread of disease and general condition of the patient), primary upfront debulking should be offered (Colombo et al., 2019).
• The ESGO quality guidelines emphasize that surgery must be performed by a gynecologist oncologist or a trained surgeon specifically dedicated to gynecologic cancers management and provide a clear guidance on the selection of the qualified surgeons and centers (Querleu et al., 2016).
• According to ASCO guidelines, for women with a high likelihood of achieving a cytoreduction to < 1 cm (ideally to no visible disease) with acceptable morbidity, primary cytoreductive surgery is recommended over neoadjuvant chemotherapy (Wright et al., 2016).
• The BGCS focuses on the importance of surgical resection of all visible disease and that the treatment should be planned by a specialist surgeon core MDT member in specialist centers (Fotopoulou et al., 2017).

Criteria to consider neoadjuvant chemotherapy followed by interval debulking surgery are stated in the NCCN for patients who are not good candidates for primary debulking surgery (advanced age, frailty, poor performance status, comorbidities) and for a disease unlikely to be optimally cytoreduced (Armstrong et al., 2021). In reference to the ESMO, it must be offered to patients with poor performance status, low albumin levels or very extensive tumor dissemination (Ledermann et al., 2013).

All panelists agreed on the aforementioned guidelines and on the importance of complete cytoreduction. Upfront surgery should be favored when feasible and exploratory laparoscopic surgery by expert surgeons constitutes an option when uncertainty prevails in selected patients. For primary unresectable disease or for ineligible patients due to poor general condition, three cycles of chemotherapy followed by interval surgery are recommended.

• Primary cytoreductive surgery by expert surgeons (gynecologist oncologist trained in ovarian cancer surgery) is recommended whenever feasible.
• Expert surgeon evaluation is essential and the use of laparoscopy in selected patients is advisable.
• Neoadjuvant chemotherapy followed by interval surgery is recommended alternatively for unresectable disease or ineligible patients.

Noteworthy, minimally invasive surgery (notably laparoscopic and robotic techniques) for ovarian tumor removal has been employed in early and advanced ovarian cancer cases (upfront and upon relapse); but more prospective studies are required to demonstrate their safety and efficacy (Lim et al., 2021, Nezhat et al., 2013).

#### 3.2.2 Adjuvant treatment

Platinum-based chemotherapy (mainly carboplatin) with paclitaxel has been the mainstay of HGSE ovarian cancer treatment for few decades, with dosing regimens refined over time (Lheureux et al., 2019, Monk and Chan, 2017). The current standard of care is carboplatin (AUC = 5–6) and paclitaxel (175 mg/m^2^) every three weeks for 6 cycles (Lheureux et al., 2019). For advanced ovarian cancer, chemotherapy should be received after surgery. If a neoadjuvant approach followed by interval debulking was adopted, adjuvant chemotherapy should always be added with a minimum total of 6 cycles including 3 cycles of adjuvant chemotherapy following the interval debulking surgery (Armstrong et al., 2021). Surgical outcomes, patient’s status and cumulative toxicity risks should be taken into consideration. Different formulations were evaluated and included in the recommendations, mainly intravenous, with a role of intraperitoneal chemotherapy in selected cases (Armstrong et al., 2021). The advent of targeted therapy and global concerted efforts culminated in the introduction of targeted therapies, such as bevacizumab, a humanized antibody against the vascular endothelial growth factor A (VEGF-A), thus inhibiting angiogenesis and limiting tumor growth (Ferrara and Adamis, 2016, Garcia et al., 2020). Bevacizumab is currently a pillar in first-line HGSE ovarian cancer treatment, owing to extensive research (Ferrara and Adamis, 2016, Garcia et al., 2020). Its uses are variable since it can be added selectively as neoadjuvant (with caution due to the potential healing interference (Haunschild and Tewari, 2020) and as adjuvant therapy in combination with the standard carboplatin/paclitaxel followed by maintenance bevacizumab in monotherapy. To note, to be given as maintenance monotherapy, bevacizumab should have been prescribed as adjuvant treatment in combination with carboplatin/paclitaxel first for 6 cycles with evidence of disease stability or regression upon assessment (Burger et al., 2011). In 2021, Pfisterer et al. recommended that maintenance bevacizumab be given for 15 months, based on the results of the ENGOT/GCIG trial (Pfisterer et al., 2021). These guidelines are based on the results of the ICON-7 (Oza et al., 2015) and GOG-218 (Ferriss et al., 2015) trials where different dosage administration of bevacizumab were evaluated (7.5 mg/kg in ICON-7 and 15 mg/kg in GOG-218) as well as different population inclusion criteria (stage I–IIA clear-cell/grade 3 or stage IIB/IV in ICON-7 and Stage III and IV in GOG-218). Both of these studies showed a significant progression-free survival (PFS) benefit of adding bevacizumab in combination with carboplatin/paclitaxel followed by maintenance bevacizumab (PFS hazard ratio [HR] = 0.77, with 95% confidence interval [CI] 0.59–0.99 in ICON-7 (González Martín et al., 2019) and HR = 0.717, with 95% CI 0.625–0.824 in GOG-218 (Burger et al., 2011) with no significant overall survival benefit after long-term follow-up. However, when post-hoc subgroup analysis was performed, both studies revealed significant overall survival benefits in patients with poor prognostic features (stage IV and ascites in GOG-218 and patients with stage III with ˃ 1 cm residual disease, stage IV or inoperable disease in ICON-7) (Tewari et al., 2019).

Additionally, the use of hyperthermic intraperitoneal chemotherapy (HIPEC) with interval cytoreductive surgery might confer PFS advantage in patients with stage III epithelial ovarian cancer (van Driel et al., 2018, van Stein et al., 2021). However, data on the benefits of HIPEC remains inconsistent, and more trials need to be undertaken to standardize the use of HIPEC (dose and timing) and to describe definitive benefits and adverse effects (Tsuyoshi et al., 2020, Vergote et al., 2019).

Panelists discussed scientific data as well as the practical implication of using bevacizumab in advanced ovarian cancer and they shared their real world experience. Some of them prefer its use only in high risk patients (stage IV and Stage III with residual disease) while others prefer to use it for all advanced ovarian cancer patients. In practice, in Lebanon, patients possess different coverage parties and this also affects their practice with some coverage systems, including the Ministry of Public Health, reimbursing bevacizumab only for high risk patients.

#### 3.2.3 Maintenance therapy

In a paradigm shift, maintenance therapy has now become vital in patients with advanced HGSE ovarian cancer, since despite initial response to first-line treatment, most patients relapse within three years of treatment completion (Ledermann et al., 2013). In addition to anti-angiogenic therapy with bevacizumab, novel maintenance approaches are currently based on molecular pathways for DNA repair. Through impeding DNA repair, PARP inhibitors proved effective in prolonging the survival of patients with recurrent disease (Coleman et al., 2017, Mirza et al., 2016), motivating their re-evaluation as first-line maintenance treatment, with the hope of preventing or delaying relapse. Four trials evaluated different PARP inhibitors as maintenance therapy in newly diagnosed advanced ovarian cancer**.** These trials changed the face of first-line treatment of advanced HGSE ovarian cancer and shifted the paradigm towards molecular targeting of pathways involved in cancer progression.

Table 1 provides an overview of the trials summarizing the clinical research on PARP inhibitor use for first-line maintenance therapy in newly diagnosed advanced HGSE ovarian cancer.

**Table 1:** PARP inhibitors in advanced HGSE ovarian cancer.

	**SOLO-1 (10)** **Phase 3** **2018**	**PAOLA-1 (11)** **Phase 3** **2019**	**PRIMA (12)** **Phase 3** **2019**	**VELIA (13)** **Phase 3** **2019**
**Trial design**	International, randomized, double-blind	International, randomized, double-blind	Randomized, double-blind	International, randomized, double-blind
**Number of patients**	391	806	733*	1140
**BRCA status**	Mutated	Wild-type or Mutated	Wild-type or Mutated	Wild-type or Mutated
**HRD status**	Unknown	Positive (N = 387, 48.0%) or Negative	Positive (N = 373, 50.9%) or Negative	Positive (N = 627, 55.0%) or Negative
**Bevacizumab**	No	Yes	No**	No
**Treatment arms**	2:1 Olaparib *versus* Placebo	2:1 Olaparib + bevacizumab *versus* Placebo + bevacizumab	2:1 Niraparib *versus* Placebo	1:1:1 *** Chemotherapy + placebo followed by maintenance placebo *versus* Chemotherapy + Veliparib followed by maintenance placebo *versus* Chemotherapy + Veliparib followed by maintenance veliparib
**Primary outcome**	Progression-free survival	Disease progression or death	Progression-free survival	Progression-free survival
**Main result**	HR = 0.33*P* < 0.001	Overall HR = 0.59*P* < 0.0001 HRD^+^ (including *BRCAm*) HR = 0.33*P* < 0.0001 HRD+ (excluding *BRCAm*) HR = 0.43*P* < 0.0001 HRD^-^ (i.e. HRP) HR = 0.92	Overall HR = 0.62*P* < 0.001 HRD^+^ (including *BRCAm*) HR = 0.43*P* < 0.001 HRD^+^ (excluding *BRCAm*) HR = 0.50*P* < 0.001 HRD^-^ (i.e. HRP) HR = 0.68	Overall HR = 0.68*P* < 0.001 HRD^+^ HR = 0.57*P* < 0.001 BRCAm^+^ HR = 0.44*P* < 0.001
BRCA: breast cancer gene; HR: hazard ratio; HRD: homologous recombination deficiency; HRP: homologous recombination proficiency *Patients with Stage III ovarian cancer and no residual disease were excluded. Only patients at high risk for treatment failure were included. **Bevacizumab was not yet approved for first-line maintenance treatment. ***Veliparib or placebo were given as induction treatment with chemotherapy, and then followed with maintenance therapy with veliparib or placebo.				

In all four trials, there were no statistically significant differences between treatment arms in the heath-related quality of life metrics evaluated. To note, the PRIMA trial excluded Stage III patients who had complete cytoreduction while the PAOLA-1 trial included Stage III and Stage IV patients regardless of surgical outcomes.

### 3.3 International recommendations on the use of PARP inhibitors in maintenance therapy

Given the robustness of clinical trial outcomes, the treatment paradigm shift for ovarian cancer was reflected by updated treatment recommendations laid down by leading Medical Societies concerned with ovarian cancer.

Table 2 details an algorithmic scheme of ESMO and NCCN first-line maintenance guidelines.

**Table 2:** First-line maintenance therapy, according to ESMO (15, 44) and NCCN (22) guidelines.

**NCCN**	**ESMO**
Patients with stages II-IV ovarian cancer post primary treatment, ***who did not have bevacizumab*** during primary therapy and have: 1. Wild-type or unknown BRCA status, showing complete or partial response, should be monitored or given niraparib as maintenance therapy 2. Germline or somatic BRCA mutation, showing complete or partial response, should be given olaparib or niraparib; stage II ovarian cancer could be monitored	Patients with a BRCA mutation and a partial or complete response to front-line platinum-based chemotherapy should receive maintenance treatment with a PARPi: • Olaparib for 2 years (ESMO-MCBS score of 4) • Niraparib for 3 years (ESMO-MCBS score 3) • Olaparib + bevacizumab if bevacizumab is given with chemotherapy (ESMO-MCBS score 3)
Patients with stages II-IV ovarian cancer post primary treatment, ***who did have bevacizumab*** during primary therapy and have: 1. Wild-type or unknown BRCA status, showing complete or partial response, and who are HRP or with unknown status should be given bevacizumab 2. Wild-type or unknown BRCA status, showing complete or partial response, and who are HRD^+^ should be given bevacizumab together with olaparib 3. Germline or somatic BRCA mutation, showing complete or partial response can be given bevacizumab with olaparib, or olaparib alone or niraparib alone	Newly diagnosed advanced ovarian cancer patients, who are HRD^+^ and with partial and complete response to chemotherapy should receive a PARPi (olaparib + bevacizumab if started with chemotherapy or niraparib).
Patients with stable or recurring disease need to be managed differently	
BRCA: breast cancer gene; ESMO: European Society of Medical Oncology; MCBS: Magnitude of clinical Benefit Scale; NCCN: National Comprehensive Cancer Network; PARP: poly(ADP-ribose) polymerase; PARPi: PARP inhibitor	

#### 3.3.1 Currently applicable guidelines in Lebanon

As of 2018, advanced cases are treated with aggressive surgical debulking surgery and standard chemotherapy (paclitaxel and carboplatin, every 3 weeks for 6 cycles). The national cancer guidelines also shed the light in 2018 on newly evolving standard of care involving intraperitoneal chemotherapy with paclitaxel and cisplatin every 3 weeks in patients with small-volume residual disease (Committee, 2018).

#### 3.3.2 Recommendation for local practice

Panelists reviewed the trials and discussed the international guidelines and algorithms. They drew attention to the difference in the eligibility criteria between the PRIMA and PAOLA-1 trials as well as the fact that in PRIMA, the comparator arm was placebo while in PAOLA-1, it was bevacizumab plus placebo; thus affecting the interpretation and comparison of results. They all agreed on the role of PARP inhibitors as an essential pillar standard of care maintenance therapy in advanced ovarian cancer. They recommend early testing for all new patients. In addition, Olaparib was the main PARP inhibitor discussed, since it is currently the only one approved and available in Lebanon.

• Some panelists prefer the combination of bevacizumab plus Olaparib over Olaparib monotherapy as maintenance and thus recommend performing early HRD testing and adding bevacizumab to adjuvant chemotherapy for all patients. Thus, according to the results of HRD testing, maintenance with Olaparib + bevacizumab is recommended for HRD^+^ patients.
• Remaining panelists prefer Olaparib monotherapy for *BRCAm* patients and thus recommend early HRD or BRCA testing. In case of BRCAm^+^ or HRD^+^/BRCAm^+^, Olaparib monotherapy is preferred. Patients who underwent BRCA testing and turned out not to harbor the BRCA mutation are advised to undergo HRD testing, given that about 20% might still benefit from a combination maintenance therapy. The combination also is recommended for patients who had upfront HRD testing and found to be HRD^+^/BRCA^-^.

These recommendations only apply to patients with complete or partial response to treatment.

The safety profile of PARP inhibitors and the difference in the occurrence of common side effects between different PARP inhibitors was discussed by the panel as well as the management of these events. Overall, the perception is that PARP inhibitors present a relatively tolerable and manageable safety profile. These events are managed mainly by dose reduction and, in some cases by dose interruptions, without compromising clinical efficacy.

• PARP inhibitors are recommended as a standard of care maintenance therapy to targeted advanced ovarian cancer patients who respond partially or completely to surgery + chemotherapy.
• Olaparib is the only current PARP inhibitor available and approved in Lebanon for ovarian cancer.
• For BRCA mutated patients or HRD^+^/BRCA^+^ patients, it can be prescribed as maintenance monotherapy or in combination with bevacizumab.
• For HRD^+^/BRCA^-^ patients, the only approved indication is the combination with bevacizumab.
• PARP inhibitors have an acceptable, tolerable and manageable side effects profile.
• Early testing is advisable and helps dictate treatment decisions.

## 4 Genetic testing

### 4.1 BRCA testing

It has been shown that around 10–15% of epithelial ovarian cancer (EOC) patients carry germline mutation in BRCA1 or BRCA2 with the highest incidence seen with HGSE subtypes (Manchana et al., 2019). These constitute inherited mutations. However, BRCA mutations can be acquired as well, accounting for somatic mutations. It is also noteworthy to relate that both males and females may harbor germline mutations in the *BRCA* genes as this is relevant in terms of genetic history assessment through genetic counselling (Neff et al., 2017). BRCA proteins play a key role in DNA damage repair and identifying BRCA mutations has an impact on screening and prevention but also on prognosis. It also permits prediction of response to platinum-based chemotherapy and to PARP inhibitors (Swisher et al., 2017). Hence, in reference to the implications for prevention, for treatment and for ovarian cancer risk determination, international guidelines have been set:

• The NCCN recommends that all women diagnosed with epithelial ovarian, fallopian tube, and/or peritoneal cancers should be offered genetic counselling and testing for germline and somatic BRCA1/2 mutations (Armstrong et al., 2021).
• The ESMO guidelines state that all patients with high-grade ovarian cancer should be tested for BRCA1 and BRCA2 mutations (germline/somatic) at diagnosis (Colombo and Ledermann, 2021).
• ASCO guidelines conclude that all women with epithelial ovarian cancer should have germline genetic testing for BRCA1/2 and other ovarian cancer susceptibility genes. In women who do not carry a germline pathogenic or likely pathogenic BRCA1/2 variant, somatic tumor testing should be performed (Konstantinopoulos et al., 2020).
• The BGCS refers for parallel tumor and germline testing for BRCA1 and BRCA2 with a preference to offer testing as early in the patient journey as possible (Sundar et al., 2021).

### 4.2 HRD testing

Testing for HRD comprises tissue testing for BRCA mutations in addition to genomic instability assays or scores (Ngoi and Tan, 2021, Stewart et al., 2022). A positive HRD test is thus a result of a direct BRCA mutation and/or genetic or epigenetic mutations leading to a HRD, compromising a key pathway in the DNA damage repair system, a hallmark of cancer. Noteworthy, some patients with HRD-positive tumors might harbor wild-type BRCA genes (Ray-Coquard et al., 2019), but have other defects in the DNA damage repair pathway, such as loss of heterozygosity (LOH), telomeric allelic imbalance (TAI) and large scale state transitions (LST), commonly known as genomic scars (Wagener-Ryczek et al., 2021, Watkins et al., 2014). These markers for HRD are evaluated using single nucleotide polymorphism (SNP) arrays (Wagener-Ryczek et al., 2021), and their presence separately or collectively hold the potential to predict response to PARP inhibitors beyond BRCA status (Hodgson et al., 2018).

The PRIMA (González-Martín et al., 2019) and PAOLA-1 (Ray-Coquard et al., 2019) trials both have shown that about 50% of patients with ovarian cancer harbor a HRD. These include around 30% BRCA mutations (somatic or germline) and 20% non-BRCA mutated HRD^+^ due to high genomic instability scores. The presence of either or both components expresses an inability of the cell to repair double strand DNA breaks. HRD also constitutes a molecular biomarker allowing prediction of response to PARP inhibitors; for clinical decision making (Colombo and Ledermann, 2021). To note, the definition and quantification of genomic instability is heterogeneous between laboratories.

HRD testing is relatively a new concept and not all societies have therefore included it in their guidelines yet.

• According to the NCCN, there is a uniform consensus regarding the use of HRD testing to inform maintenance therapy selection following first-line chemotherapy. NCCN members disagree on the timing of testing; some members recommend testing only as needed while others recommend it as early as possible in the disease course (Armstrong et al., 2021).
• ESMO updated guidelines recommend testing for genomic instability (HRD). HRD tests that incorporate scores of allelic imbalance (Genomic Instability Score or Loss of Heterozygosity) identify a subgroup of wild-type BRCA, platinum-sensitive cancers that derive a greater magnitude of benefit from PARP inhibitor therapy (Colombo and Ledermann, 2021).
• ASCO guidelines state that no recommendations can be made yet supporting routine tumor testing using currently available HRD assays (Konstantinopoulos et al., 2020).

#### 4.2.1 Recommendation for local practice

All panelists uniformly recommend genetic testing for ovarian cancer patients and emphasize the clinical importance of early testing. BRCA and HRD tests are complementary. However they have different perceptions of the type and sequence of testing:

• Some members prefer recommending HRD testing upfront. They also recommend parallel germline BRCA testing for genetic counselling of the patient herself and her family.
• Others prefer sequential testing with BRCA testing first then HRD testing when the BRCA mutation test is negative. BRCA testing can be done on tissue upfront or sequentially starting with germline first then on tissue if the germline BRCA turns out negative. If BRCA mutation test was performed on tissue and positive, they recommend reflex germline BRCA testing for genetic counselling purposes since tissue testing does not inform on the origin of the mutation (germline *versus* somatic). With sequential testing, timing and turnover times might constitute a limitation.

Major limitations to testing in Lebanon include the cost of the test and the fact that testing and treatment are both not reimbursed. Moreover, tissue testing is not yet performed in Lebanon and samples have to be prepared and sent abroad.

• Refer all ovarian cancer patients for early genetic testing.
• BRCA and HRD testing are complementary.
• The sequence of testing (HRD upfront or BRCA first then HRD when BRCA mutation is negative) are both applicable.
• Upfront or reflex germline BRCA testing is indicated for genetic counselling and assessment.

## 5 Management of platinum sensitive relapsed ovarian cancer

### 5.1 Role of MDT and secondary surgery

Platinum-sensitive disease refers to cases that remain in remission for at least six months after the completion of the initial platinum-based treatment; i.e. when disease recurs beyond a 6-month treatment-free interval (Baert et al., 2021) The evaluation of these patients is preferably done within the core of a MDT that assesses the status of patients, their fitness to chemotherapy, previous regimens, toxicity and response, the degree of progression, and symptomatology (Colombo et al., 2019). In addition, the weight of benefits against risks of a secondary debulking surgery should be discussed with a gynecologist oncologic surgeon and more importantly the predicted expectancy of a complete debulking surgery. Hence, secondary debulking surgery can be offered to a subset of patients with limited cancer spread, for whom a complete resection can be achieved and who have a good performance status (Ledermann et al., 2013). As recommended by both ESMO and NCCN, secondary surgery can be recommended to selected patients and has to be performed in specialized centers. The DESKTOP III (Harter et al., 2006) showed that secondary cytoreductive surgery offers survival benefits in platinum-sensitive relapsed cases for patients where zero residual disease can be reached.

Panelists agree that expert surgeons and MDT evaluation are recommended for platinum-sensitive relapse. In addition to all the factors mentioned above, Arbeitsgemeinschaft Gynaekologische Onkologie (AGO) scores should be calculated; patients with a positive AGO score (Eastern Cooperative Oncology Group Performance Status [PS ECOG] 0, ascites ≤ 500 ml, and complete resection at initial surgery) benefited from secondary cytoreductive surgery (Du Bois et al., 2020). Additionally, secondary debulking surgery is only an option when complete resection (R0) is predicted (Jiang and Li, 2021). Patients should be informed about the expected outcomes, complications and the survival benefits in correlation with residual disease.

### 5.2 Role of bevacizumab

Both the OCEANS (Aghajanian et al., 2012) and the GOG0213 (Coleman et al., 2017) trials revealed an increase in PFS with the addition of bevacizumab to platinum-based chemotherapy (carboplatin or cisplatin). According to the ESMO p (Colombo et al., 2019) and the NCCN (Armstrong et al., 2021), bevacizumab in combination with second-line platinum-based chemotherapy followed by bevacizumab maintenance has proven benefit with respect to tumor response rate and PFS, and could be recommended. The NCCN does not recommend the addition of bevacizumab for patients with a risk of gastrointestinal perforation and notify that there is limited data on the use of bevacizumab in patients previously treated with it.

### 5.3 Maintenance therapy with PARP inhibitors

Several trials showcased a survival benefit of maintenance with PARP inhibitors after response to platinum-based therapy in recurrent platinum-sensitive ovarian cancer. In the NOVA (Mirza et al., 2016) and NORA (Wu et al., 2021) trials, Niraparib was evaluated as second-line maintenance treatment. Niraparib maintenance treatment reduces the risk of disease progression or death and prolongs PFS in patients with recurrent ovarian. The NOVA trial resulted in a significantly longer PFS, regardless of the presence or absence of g*BRCA* mutations or HRD status, with moderate bone marrow toxicity. The ARIEL3 study showed that Rucaparib improved PFS (Coleman et al., 2017), irrespective of progression-free interval following penultimate platinum, number of lines of prior chemotherapy, and previous use of bevacizumab (Clamp et al., 2021). Study 19 evaluated the use of Olaparib against placebo in patients with platinum sensitive relapse who respond to platinum rechallenge (Poveda et al., 2021). Results revealed a statistically significant PFS benefit in all patients with a more pronounced benefit for BRCA-mutated patients and an overall survival clinically meaningful benefit. SOLO-2 selected only BRCA-mutated patients and showed statistically significant benefit in PFS and overall survival. It evidenced a 13-month improvement in overall survival.

Table 3 reviews treatment algorithms proposed by the NCCS and the ESMO in case of platinum-sensitive relapse.

**Table 3:** NCCN (Armstrong et al., 2021), ESMO (Colombo et al., 2019, Ledermann et al., 2013) and ASCO (Bouberhan et al., 2019) treatment algorithms in platinum-sensitive relapsed patients.

**NCCN**	Olaparib as maintenance therapy for women with ovarian cancer who have received 2 or more lines of chemotherapy. (Preferred for BRCA mutations).
**ESMO**	PARP inhibitors (olaparib, niraparib and rucaparib) when given as maintenance therapy following a response to platinum-based second or higher line of treatment have proven benefit with respect to progression-free survival and could be recommended. The benefit is greatest in, but is not limited to, patients with a BRCA mutation.
**ASCO**	Maintenance with PARP inhibitors is preferred for patients with a germline or somatic BRCA mutation, who achieved complete or partial remission with platinum based chemotherapy. Those with wild-type BRCA might benefit from either PARP inhibitor or bevacizumab maintenance therapy
ASCO: American Society of Clinical Oncology; BRCA: breast cancer gene; ESMO: European Society of Medical Oncology; NCCN: National Comprehensive Cancer Network; PARP: poly(ADP-ribose) polymerase; PARPi: PARP inhibitor.	

In summary, PARP inhibitors (Olaparib, Niraparib and Rucaparib) have been shown to benefit patients when given as maintenance therapy following a response to platinum-based treatment for platinum sensitive relapse and thus are recommended. The benefit includes all patients regardless of biomarker status. Nevertheless, it is clearly higher for patients with a BRCA mutation.

Although evidence on the beneficial use of PARP inhibitors in platinum-resistant (cases progressing or relapsing after platinum-based chemotherapy (Flynn and Ledermann, 2022) recurrent ovarian cancer is building up (Agarwal et al., 2021, Vanderstichele et al., 2019), the maximal benefit of PARP inhibitor use is still expected among relapsed cases who are sensitive to platinum-based chemotherapy.

#### 5.3.1 Currently applicable guidelines in Lebanon

As of 2018, treatment of platinum-resistant recurrent cases includes pegylated liposomal doxorubicin, topotecan or gemcitabine. Platinum-sensitive cases are offered either paclitaxel/carboplatin, pegylated liposomal doxorubicin/carboplatin, or gemcitabine/carboplatin (Committee, 2018).

#### 5.3.2 Recommendation for local practice

Panelists uniformly agree on the survival benefits of PARP inhibitors as maintenance therapy for patients who respond to platinum-based chemotherapy. Benefits are more pronounced in BRCA mutated patients however testing is not needed since all patients benefit from PARP inhibitor maintenance in this setting. Testing for germline BRCA mutations can be done for genetic counselling purposes. In fact, platinum sensitivity is by itself a biomarker for predicted sensitivity and response to PARP inhibitors.

• PARP inhibitors are recommended as maintenance therapy for platinum-sensitive relapsed patients who respond to platinum based chemotherapy regardless of BRCA mutation or HRD status.
• Testing for germline BRCA mutations can be done for genetic counselling purposes.

## 6 Conclusion

The management of ovarian cancer in general, and of advanced HGSE ovarian cancer in particular, should be discussed through and dictated by a MDT and decisions on treatment should be taken on a case-by-case basis. A nationwide monthly tumor board that includes an oncologist, a radiologist, a pathologist and an expert gynaecologic cancer surgeon is highly recommended. Newly diagnosed advanced HGSE ovarian cancer is ideally treated by upfront complete cytoreductive surgery, followed by adjuvant platinum-based chemotherapy (carboplatin) with paclitaxel with or without bevacizumab, and complemented by a maintenance therapy. Neoadjuvant chemotherapy followed by interval surgery is an alternative that can be offered to selected patients. Maintenance therapy has a prominent role in ovarian cancer since most patients relapse after first-line treatment and bevacizumab or PARP inhibitors are the approved maintenance therapy options. PARP inhibitors’ trials showcased unprecedented survival benefits in monotherapy or in combination with bevacizumab (for Olaparib) especially for biomarker selected patients. Hence early genetic testing is recommended. For platinum-sensitive relapsed ovarian cancer, a MDT is essential, and secondary debulking surgery is indicated only if zero residual disease is predicable. Platinum chemotherapy rechallenge is primordial and the addition of bevacizumab might be beneficial. PARP inhibitors for maintenance treatment to patients with response are recommended regardless of BRCA mutation or HRD status.

The Lebanese local practice needs updating at the level of the Ministry of Public Health. A task force (including authors of the current work, other experts in ovarian cancer management in Lebanon, and other stakeholders) for updating the national cancer treatment guidelines needs to be developed. This task force will also evaluate the implementation of these practice guidelines and oversee data collection in the ongoing National Cancer Registry at the Ministry of Public Health. However, the current work is initially aimed and hoped to serve as a practical guidance for the management of patients with advanced and platinum-sensitive newly diagnosed and relapsed ovarian cancer based on state-of-the-art evidence; to subsequently aid for the endorsement of updated recommendations by all concerned stakeholders in the management of ovarian cancer.

## CRediT authorship contribution statement

**Reem Abdallah:** Conceptualization, Methodology, Supervision, Funding acquisition, Writing – original draft, Writing – review & editing. **David Atallah:** Writing – original draft, Writing – review & editing. **Nizar Bitar:** Writing – original draft, Writing – review & editing. **Georges Chahine:** Writing – original draft, Writing – review & editing. **Hady Ghanem:** Writing – original draft, Writing – review & editing. **Marwan Ghosn:** Writing – original draft, Writing – review & editing. **Joseph Kattan:** Writing – original draft, Writing – review & editing. **Fadi Nasr:** Writing – original draft, Writing – review & editing. **Joseph Makdessi:** Writing – original draft, Writing – review & editing. **Ali Shamseddine:** Writing – original draft, Writing – review & editing.

## Declaration of Competing Interest

The authors declare that they have no known competing financial interests or personal relationships that could have appeared to influence the work reported in this paper.
